# Doctor Rudolph Beck

**Published:** 1913-05-15

**Authors:** 


DOCTOR RUDOLPH BECK. Dr. Beck died March 15th at his home in Chicago, at the age of 44, from a brain tumor. Dr. Beck was well-known in Chicago and the Middle West because of his active relations to professional work in the various society organizations. He perhaps is better known for the advocacy of a paste of Bismuth advocated by his brother—a medical man— for surgical dressings. Dr. Beck's application of this paste to the treatment of dental necrotic conditions met with very profitable results. He was a good professional man and highly esteemed by all who knew him.

